# Risk factors for drug-related acute pancreatitis: an analysis of the FDA adverse event reporting system (FAERS)

**DOI:** 10.3389/fphar.2023.1231320

**Published:** 2023-11-17

**Authors:** Lin Zhang, Wei Mao, Dan Liu, Bin Hu, Xiaofang Lin, Jie Ran, Xingxing Li, Jing Hu

**Affiliations:** ^1^ Department of Pharmacy, The first Affiliated Hospital of Army Medical University (Third Military Medical University), Chongqing, China; ^2^ Department of Pharmacy, Nanan People’s Hospital of Chongqing, Chongqing, China

**Keywords:** acute pancreatitis, adverse events, pharmacovigilance, FAERS, gender

## Abstract

**Objective:** While several drugs have been linked to acute pancreatitis (AP), the AP-related risk of most drugs remains unclear. This study investigated the risk factors for drug-induced AP by analyzing a large dataset from the FDA Adverse Event Reporting System (FAERS).

**Methods:** The reporting odds ratios (ROR) were used to assess the reports of drug-induced AP from the first quarter of 2004 to the second quarter of 2022. Single-factor, LASSO, and multi-factor regression analysis were performed to explore drug-related AP-related risk factors. Bonferroni correction was applied for the multiple comparisons performed.

**Results:** A total of 264 drugs associated with AP, including antineoplastic drugs (35/264), antidiabetic drugs (28/264), antibacterial drugs (24/264), immunomodulatory drugs (11/264), antipsychotic drugs (6/264), and other drugs (160/264) were retrieved. Multi-factor analysis showed that males, age 41–54 years old, and 36 drugs, including Tigecycline, were risk factors for drug-related AP. The median time to drug-related AP onset was 31 days (interquartile range [IQR] 7–102 days) and about 75% of adverse events occurred within 100 days.

**Conclusion:** These findings may help clinicians to identify drug-related AP at the early stage and can be used to inform future studies of drug-related AP pathogenesis.

## 1 Introduction

AP is an inflammatory disease of the pancreas associated with the sudden onset of persistent abdominal pain, nausea, vomiting, fever, and abdominal distention, and is the leading cause of gastrointestinal disorder-related hospitalizations worldwide (Lankisch et al., 2015). AP can cause multiple organ dysfunction that results in pancreatic necrosis and persistent organ failure, with an associated mortality rate of 1%–5% (Garg and Singh, 2019; Szatmary et al., 2022). While global AP mortality has declined over the past decade, the incidence rate has increased to 34 cases per 100,000 people (Petrov and Yadav, 2019). AP has numerous established etiologies, of which gallstones and alcohol are the most common. Other causes include diabetes, obesity, smoking, hyperlipidemia, endoscopic retrograde cholangiopancreatography, and various medications (Lankisch et al., 2015; Matta et al., 2020; Mederos et al., 2021). While drugs only account for about 5% of AP cases (Lankisch et al., 2015), with most being mild to moderate, some have been associated with serious complications or even death (Wolfe et al., 2020). Thus, it is critical to identify medications that may be linked to this condition.

The etiological and pathological mechanism of drug-induced AP remains unclear. While most drug-induced AP cases are considered idiopathic, some studies have suggested an immune-mediated association (Badalov et al., 2007). Early diagnosis of AP and discontinuation of pathogenic drugs can reduce complications (Szatmary et al., 2022). Studies have linked more than 180 drugs to AP, however, each drug was only used for a few cases, mostly as case reports, and large-scale studies are lacking (Trivedi and Pitchumoni, 2005; Ksiądzyna, 2011; Simons-Linares et al., 2019; Wolfe et al., 2020).

Our previous study of FAERS data found that SGLT-2i can increase the risk of AP (Zhang et al., 2022). However, the association between all the drugs in FAERS and AP was not assessed. Thus, this study sought to comprehensively evaluate the risk factors related to drug-induced AP. Since FAERS is the largest database of adverse event reports (Rodriguez et al., 2001), FAERS was used to comprehensively investigate drug-induced AP and explore potential risk factors.

## 2 Materials and methods

### 2.1 Data source and collection

Data from the first quarter of 2004 to the second quarter of 2022 were downloaded from FAERS. Each file contained seven types of data, including DEMO(contains patient demographic and administrative information, a single record for each event report), REAC (contains all “Medical Dictionary for Regulatory Activities” (MedDRA) terms coded for the event), DRUG (contains drug/biologic information for as many medications as were reported for the event), OUTC(contains patient outcomes for the event), RPSR(contains report sources for event), THER(contains drug therapy start dates and end dates for the reported drugs), and INDI (contains all MedDRA terms coded for the indications for use/diagnoses for the reported drugs), and was imported into PostgreSQL. Duplicate data was deleted as recommended by the FDA. If cases had the same case ID, the most recent report with fda_dt was retained, and if the case ID and fda_dt were the same, the report with the larger primary ID was retained. A few primary ID duplicates were still found after de-duplication so a secondary de-duplication was performed. Adverse events (AEs) in REAC were coded by preferred terms (PTs) according to the MedDRA. AP included: pancreatitis necrotizing, pancreatitis acute, pancreatitis, pancreas infection, haemorrhagic necrotic pancreatitis, pancreatitis haemorrhagic, ischaemic pancreatitis, pancreatic abscess, and pancreatic phlegmon. Drugs associated with AEs were assigned different roles (primary suspect, secondary suspect, concomitant, interaction), and the case was included only if the drug was listed as a “primary suspect” of AP.

A two-by-two contingency table was constructed (Supplementary Table S1). The relationship between AP and suspected drugs was assessed with disproportionality analysis using ROR and a 95% confidence interval (CI), and the suspicious drugs with AP therapeutic effect were excluded. A risk signal was considered when the ROR and the lower limit of the corresponding 95% CI was >1, a >3. The calculation formula is as follows:

$$ROR=\frac{a/c}{b/d}ROR_{95\%CI}=e^{\ln\left(ROR\right)\pm 1.96\sqrt{\left(\frac{1}{a}+\frac{1}{b}+\frac{1}{c}+\frac{1}{d}\right)}}$$

P-adjust was the *p*-value after Fisher’s exact test and Bonferroni correction. A volcano plot was drawn with -log (p*-adjust*) as the abscissa and logROR as the ordinate.

### 2.2 Regression analysis

FAERS reports containing patient information (gender, age, weight) were extracted and only those with complete data were analyzed. Age >120 years and weight >400 kg were defined as outliers and excluded from the analysis.

A single-factor analysis for suspicious drugs was conducted using a 95% CI lower limit of ROR >1, a >100, and *p-adjust* < 0.01. Suspect drugs with *p* < 0.01 in single-factor analysis were used for least absolute shrinkage and selection operator (LASSO) regression. Multi-factor logistic regression was conducted to determine the existence of drug-related AP risk factors, using the drugs screened by LASSO combined with basic patient information as independent variables.

### 2.3 Statistical analysis

Descriptive analysis was applied to summarize and present the clinical characteristics of the patients in drug-related AP reports. Screening independent risk factors of drug-related AP by multivariate regression and Bonferroni correction. Data were analyzed using PostgreSQL (version 14.4) and R software (version 4.2.1).

## 3 Results

### 3.1 Baseline characteristics of acute pancreatitis

**FIGURE 1:**
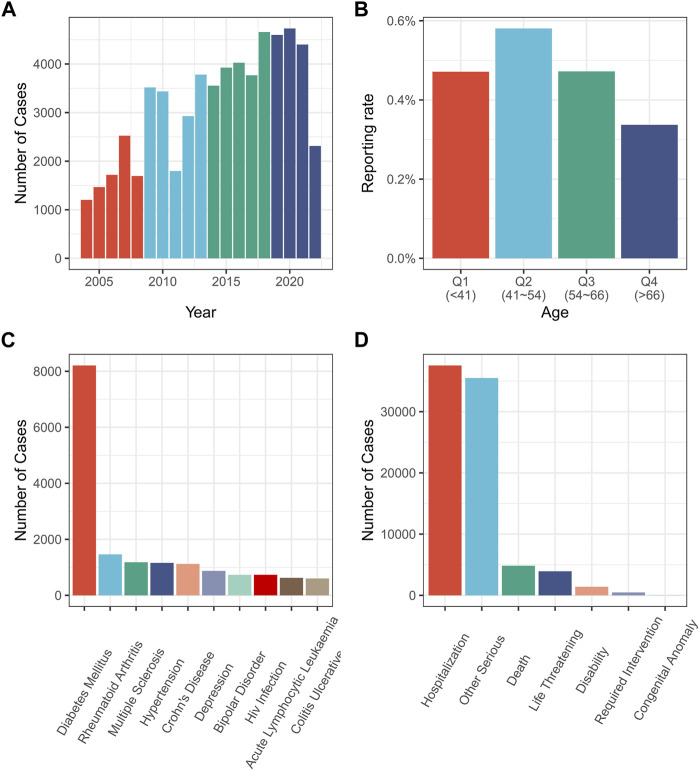
Relevant information from various drug-related AP reports. **(A)** Drug-related AP reports by year; **(B)** Reporting rate of drug-related AP by patient age; **(C)** Diagnosis of patients with drug-related AP; **(D)** Adverse reactions’ outcome with drug-related AP. AP, acute pancreatitis.

The baseline characteristics of drug-related AP are shown in Table 1 and Figure 1, and Supplementary Figure S1. A total of 14,928,209 AEs were reported from the first quarter of 2004 to the second quarter of 2022, of which 60,042 were related to AP. There were 28,696 (47.8%) reports for females, 25,408 (42.3%) reports for males and 5938 (9.9%) reports with missing gender values. The patients had a median age of 54 years (IQR 41–66 years) and a median weight of 79.4 kg (IQR 64–96.3 kg). Most (75.2%, 45163 reports) were urgent reports, and 61.9% (37163 reports) were reported by medical workers. The United States, France, and Canada reported the highest number of AP cases (21,998, 3,558, and 2,445 reports, respectively).

**TABLE 1:** Basic patient information.

Characteristics	Drug-related AP (N = 60042)
**Age**	
Median (Q1, Q3)	54.0 (41.0, 66.0)
Unknown	19296 (32.1%)
**Gender**	
Female	28696 (47.8%)
Male	25408 (42.3%)
Unknown	5,938 (9.9%)
**Weight**	
Median (Q1, Q3)	79.4 (64.0, 96.3)
Unknown	40104 (66.8%)
**The type of report submitted**	
Direct	3779 (6.3%)
Expedited (15-Day)	45163 (75.2%)
30-Day	2 (0.0%)
Periodic (Non-Expedited)	11098 (18.5%)
**Occupation of the reporter**	
Physician	20790 (34.6%)
Pharmacist	4065 (6.8%)
Other health-professional	12308 (20.5%)
Lawyer	1909 (3.2%)
Consumer	15914 (26.5%)
Unknown	5,056 (8.4%)
**Country of the reporter**	
United States	21998 (36.6%)
France	3558 (5.9%)
Canada	2445 (4.1%)
United Kingdom	1798 (3.0%)
Japan	1692 (2.8%)
Germany	1570 (2.6%)
Spain	811 (1.4%)
Italy	651 (1.1%)
Netherlands	493 (0.8%)
Brazil	446 (0.7%)
Australia	433 (0.7%)
China	267 (0.4%)
Switzerland	235 (0.4%)
Poland	214 (0.4%)
India	212 (0.4%)
Other	23219 (38.7%)

Unknown indicates for missing value. Each item included missing values; however, the analyses were performed using data without missing values; AP, acute pancreatitis.

The findings indicated that drug-related AP reports are increasing over time (Figure 1A). However, according to the age group, drug-related AP was most prevalent in patients aged 41–54 years (Figure 1B). The most frequent adverse outcome was prolonged hospitalization (Figure 1D). Diabetes accounted for the highest number of AP cases (Figure 1C). Excluding the reports with missing gender values (1,640,585 out of 14,928,209 reports reported missing gender values), drug-related AP accounted for 0.36% of the reports involving females, and 0.49% of those involving males (*p* < 0.01) (Supplementary Figure S1).

### 3.2 Drugs associated with acute pancreatitis

**FIGURE 2:**
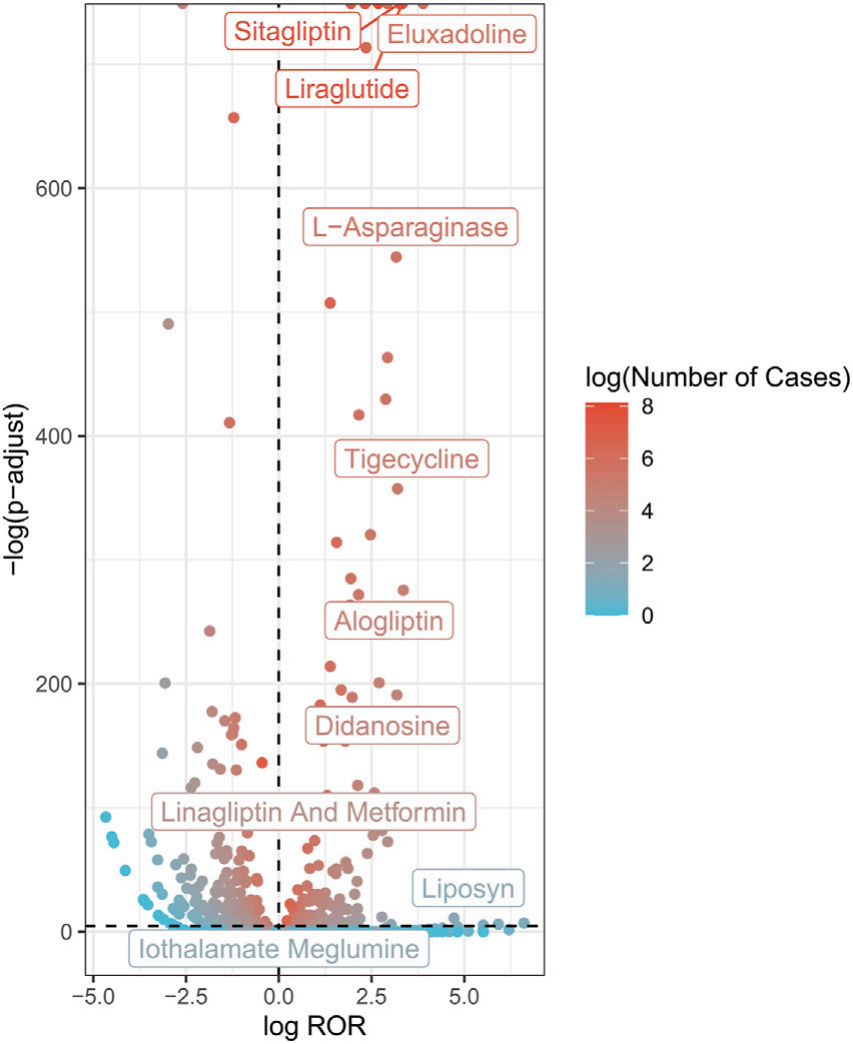
AP-related drug volcano plots. AP, acute pancreatitis; ROR, reporting odds ratio; P-adjust, *p*-value after Bonferroni correction.

Volcano plots were developed to analyze the relationship between AP and the suspected drugs (Figure 2). In each plot, the *x*-axis indicates the logarithm of the ROR. A positive x-axis indicates that adverse effects associated with drug-related AP were reported more often than other adverse effects. The *y*-axis represents the negative logarithm of the p-adjust from the *p*-value after the Fisher’s exact test and Bonferroni correction. A positive y-axis represents a strongly significant difference. The color of the dot indicates the logarithm of the number of case reports. The more red the color, the higher the number of reports. Thus, drugs in the upper right of the graph had both significant signal strength and differences.

A total of 264 AP-related drugs were identified (Supplementary Table S2). The top ten drug categories included antiviral drugs (36/264), antineoplastic drugs (35/264), antidiabetic drugs (28/264), antibacterial drugs (24/264), anti-hypertensive drugs (22/264), antiatherosclerotic drugs (13/264), analgesic drugs (13/264), immunomodulatory drugs (11/264), antipsychotic drugs (6/264), and glucocorticoids (5/264).

### 3.3 Risk factors for drug-related acute pancreatitis

**FIGURE 3:**
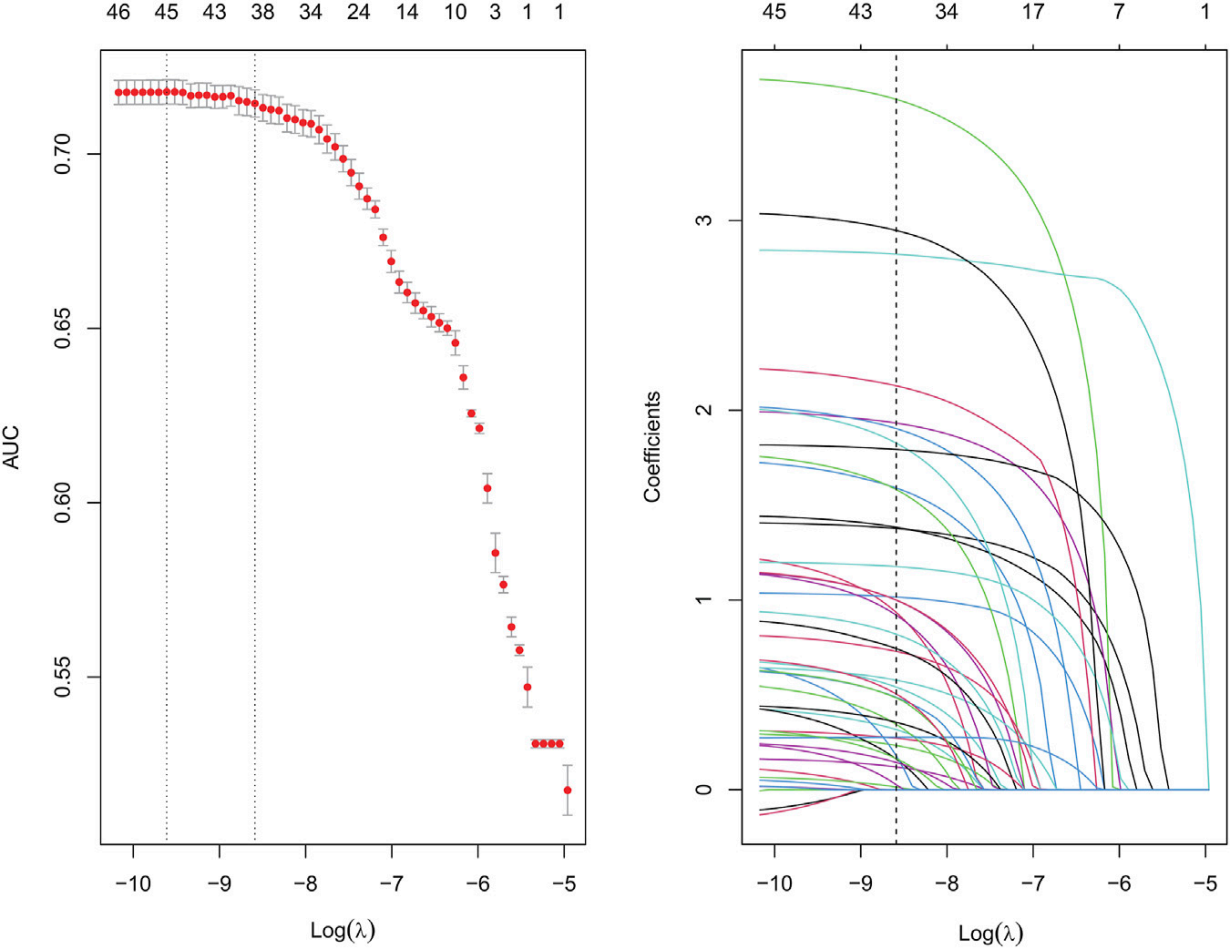
Results of the LASSO regression analysis. LASSO, least absolute shrinkage and selection operator.

**FIGURE 4:**
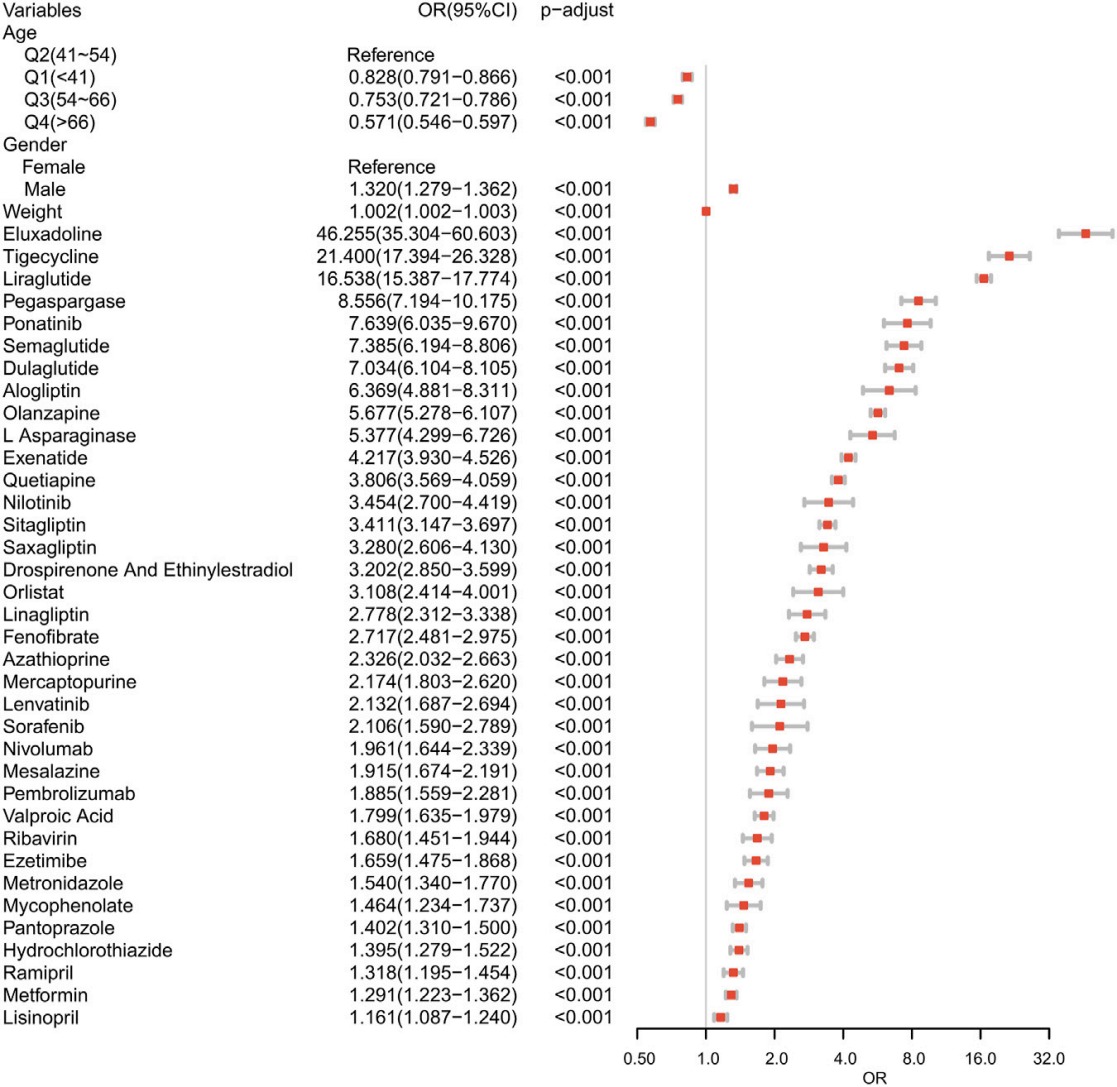
Results of the multi-factor logistic regression analysis. CI, confidence interval; OR, odds ratio; P-adjust, *p*-value after Bonferroni correction; P-adjust<0.01, statistically significant.

**FIGURE 5:**
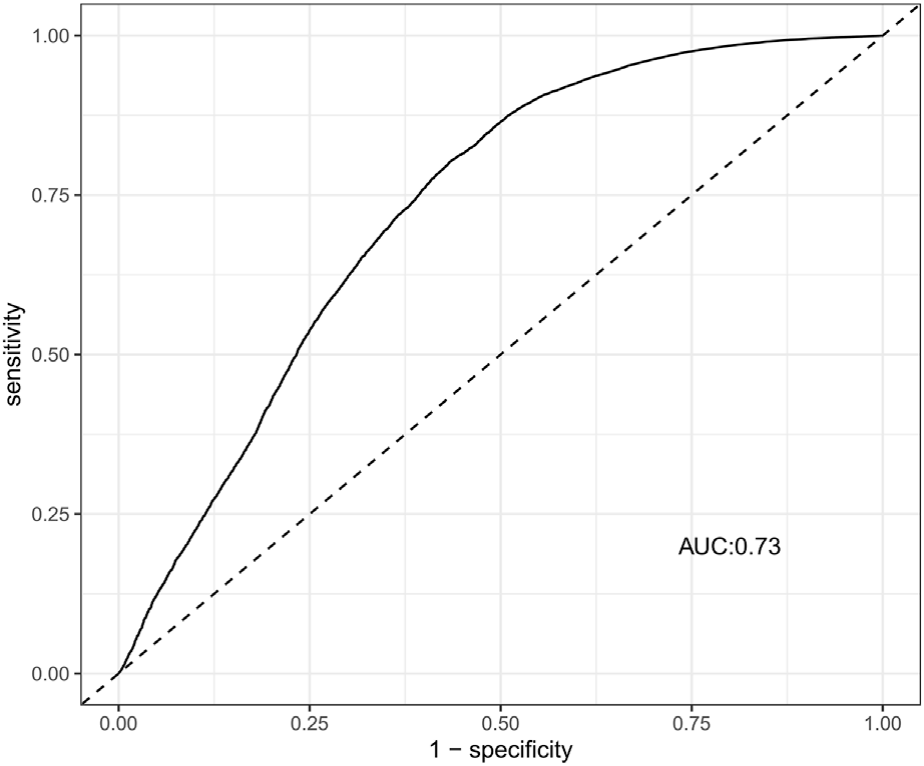
The ROC curves of drug-related AP risk factors. AP, acute pancreatitis; ROC, receiver operating characteristic; AUC, area under curve.

Suspected drugs with >100 case reports, a lower 95% CI limit of the ROR >1 and *p-adjust* < 0.01 were extracted for single-factor analysis. Drugs with *p* < 0.01 in single-factor analysis were subjected to LASSO regression, and a total of 36 drugs were screened (Figure 3). Multi-factor logistic regression analysis of these drugs was performed in combination with patient information (Figure 4). The results showed that males, age 41–54 years old, and 36 drugs including Eluxadoline, Tigecycline, Liraglutide, Pegaspargase, Ponatinib, Semaglutide, Dulaglutide, Alogliptin, Olanzapine, and others were independent risk factors of drug-related AP. The ROC-AUC, which indicates the prediction accuracy of the model, was 0.73 (Figure 5). These drugs were classified as antidiabetic (9/36), antineoplastic (8/36), immunomodulatory (3/36), anti-hypertensive (2/36), antiatherosclerotic (2/36), antipsychotic (2/36), antibacterial (2/36), diuretic (1/36), antiepileptic (1/36), gastric acid secretion inhibitor (1/36), antiviral (1/36), and other drugs (4/36) (Supplementary Table S3).

### 3.4 Time interval between drug use and the onset of acute pancreatitis

**FIGURE 6:**
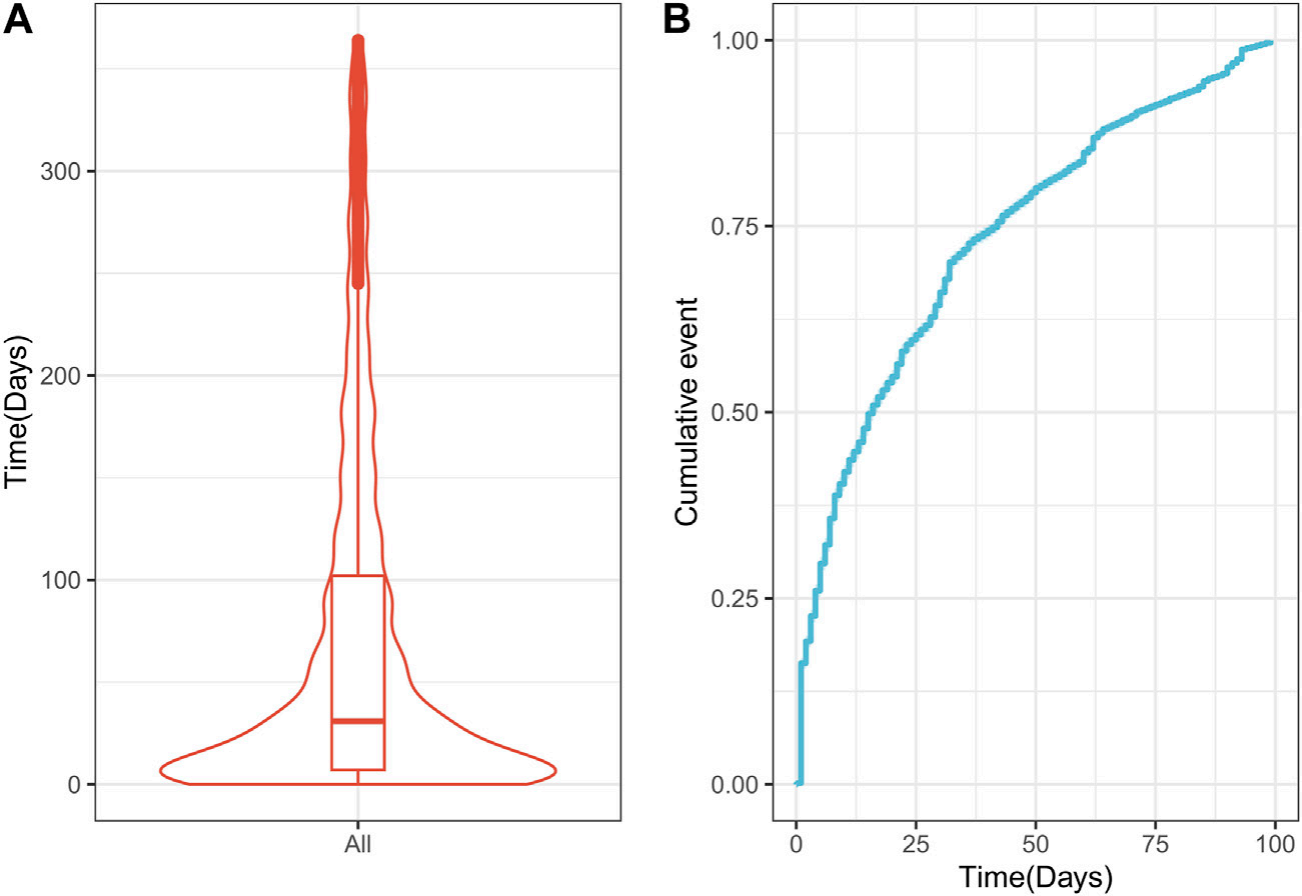
Time interval between drug Q19 intake and the onset of drug-related AP. **(A)** Violin plot of time to drug-related AP occurrence; **(B)** Cumulative incidence of drug-related AP over 100 days. AP, acute pancreatitis.

The time from drug use to AP occurrence was assessed (Figure 6). The median time to the drug-related onset of AP was 31 days (IQR 7–102 days), with approximately 75% of reported cases occurring within the first 100 days after drug initiation.

## 4 Discussion

The mechanism of drug-induced AP is unclear, and potential mechanisms include pancreatic duct constriction, cytotoxic and metabolic effects, accumulation of toxic metabolites, or hypersensitivity reactions (Jones et al., 2015). The adverse effects of drugs (e.g., causing hypertriglyceridemia and chronic hypercalcemia) have also been suggested as mechanisms for drug-induced AP (Kaurich, 2008). Since it is rare for drugs to induce AP, it has been a challenge to identify a particular medication as the cause of this condition. Numerous cases of AP linked to the use of antineoplastic (Engel et al., 2013; Chou et al., 2016; Truszkowska et al., 2022), immunosuppressive (Fontana and Cappelli, 2016; Ding et al., 2022), antiviral (Khadka et al., 2022), anti-hypertensive (Carnovale et al., 2003; Thumma et al., 2019), antidiabetic (Lee et al., 2014; Verma, 2016; Yang et al., 2016), antibacterial (Hung et al., 2009; Jiménez Moreno et al., 2020), and glucocorticoid (Yahiaoui et al., 2017; Wang-Liang et al., 2022) drugs have been reported to date. However, the evidence for an association between these drugs and AP remains weak, and there is a risk of false positives. Thus, a large-scale case study has been needed to identify suspected drugs and important drug-related AP risk factors.

FAERS, the largest global database of adverse event reports from the United States, China, the European Union, and other countries, was used to comprehensively investigate suspected AP-related drugs and risk factors. This global pharmacovigilance study of over 60,000 reports identified that the median age of drug-related AP patients was 54 years (IQR 41–66 years), which is generally consistent with previous studies of AP conducted in Europe and the United States. Nesvaderani et al. (Nesvaderani et al., 2015) retrospectively investigated 932 patients presenting with AP in Sydney and found that their median age was 50 years. Similarly, a 32-year nationwide matched-cohort study in Denmark found that the median age of AP patients was 55.8 years (Kirkegård et al., 2018). However, other studies reported a significantly different median age of patients with AP. For example, the median age of AP patients in a Mexican study was 40 years (González-González et al., 2012). A report by Malta et al. explained the potential reasons for this discrepancy (Matta et al., 2020). This study identified differences in the median age of patients with AP by geographic region. While patients in India and Latin America had a median age of 39 and 43 years, respectively, patients in Europe and North America had a median age of 58 and 52 years, respectively. Our study found that the United States had the highest number of AP reports (n = 21,998; 36.6%), followed by France (n = 3,558; 5.9%) and Canada (2,445; 4.1%). Reports were concentrated in North America and Europe, resulting in an older median age, which is consistent with prior epidemiological data. In addition, this study found that age 41–54 years old was an independent risk factor for drug-related AP, and the risk of drug-related AP was higher at <41 years old than at >54 years old. A previous retrospective study of 62 patients with AP showed that younger age was an independent risk factor for AP (Li et al., 2019), and the results of this study may help to explain our findings.

The number of drug-related AP reports for females and males was 28,696 and 25,408, respectively. Among females and males, AP accounted for 0.36% and 0.49% of adverse drug events, respectively (*p <* 0.01). Meanwhile, multivariate analysis showed that male gender was an independent risk factor for drug-related AP. Previous retrospective studies found that the incidence of AP was similar for females and males, but males had a higher incidence of chronic pancreatitis (Drake et al., 2021). Some studies have also shown that the gender distribution of AP patients differs by etiology, and alcohol-related AP is more common in males (Satoh et al., 2011; Yadav and Lowenfels, 2013). However, further clinical trials that account for potential confounding are needed to determine whether gender is a risk factor for drug-related AP.

An exhaustive multi-factor analysis of the association between AP and various drugs was conducted using FAERS and 36 medications were identified as risk factors for drug-related AP (Figure 4). Antidiabetic and antineoplastic drugs were most prevalent, followed by immunomodulatory, anti-hypertensive drugs, antibacterial, antipsychotic, and other drug types.

The antidiabetic drugs, dipeptidyl peptidase-4 (DPP-4) inhibitors (Alogliptin, Sitagliptin, Saxagliptin, Linagliptin), glucagon-like peptide-1 (GLP-1) receptor agonists (Liraglutide, Semaglutide, Dulaglutide, Exenatide) and Metformin were identified as risk factors for drug-related AP in this study. Previous controversy exists whether the above antidiabetic drugs, are associated with an increased risk of AP. Some prior studies have linked DPP-4 inhibitors (Zhang et al., 2017; Pinto et al., 2018), GLP-1 receptor agonists (Faillie et al., 2014), and Metformin (Mallick, 2004) to an increased risk of AP, but the mechanism of action remains unclear. Rouse et al. (Rouse et al., 2014) found pancreatitis to be cumulative or synergistically exacerbated by the use of GLP-1 receptor agonists on a high-fat diet in animal experiments. The sequence of injury appeared to begin with acinar cell hypertrophy, progress to proinflammatory cytokine induction, and end with pancreatic vascular injury. Sitagliptin and Exenatide are shown to increase the odds of AP by 6-fold and the odds of hospitalization by 3-fold compared with other therapies (Elashoff et al., 2011; Singh et al., 2013). A meta-analysis of large randomized controlled trials on whether the use of incretin-based therapies are associated with AP found an 82% (95% CI, 1.17–2.82) increase in the odds of developing AP with the use of these medications compared with conventional therapy (Roshanov and Dennis, 2015). However, other studies have reported contradictory results. Steinberg et al. (Steinberg et al., 2017) noted that although Liraglutide was associated with increased in serum lipase and amylase, this did not predictive of an event of subsequent AP. A meta-analysis by Chen et al. (Chen et al., 2017) showed that although the risk of AP was slightly higher with DPP-4 inhibitors in both randomized controlled studies and case-control studies, this result was not observed in cohort studies. Some other studies have even found that the use of insulin-based drugs is not associated with an increased risk of AP (Monami et al., 2011; Alves et al., 2012; Thomsen et al., 2015; Azoulay et al., 2016). More interestingly, the results of a recent network meta-analysis revealed that DPP-4 may increase the risk of AP, while GLP-1RA appears to have no effect (Kanie et al., 2021). As a result, any patient diagnosed with AP after using antidiabetic drugs should be considered for further evaluation.

Multi-factor analysis showed that protein kinase inhibitors (Ponatinib, Nilotinib, Lenvatinib, Sorafenib), immune checkpoint inhibitors (Nivolumab, Pembrolizumab), L-asparaginase and Pegaspargase were risk factors for inducing drug-related AP.

Nausea and vomiting are the most common gastrointestinal adverse effects of antineoplastic drugs, however rare instances of antineoplastic drug-related AP can occur. Pegaspargase, a covalent conjugate of polyethylene glycol (PEG), and Asparaginase are both used to treat acute lymphoblastic leukemia (ALL). Both Pegaspargase and L-asparaginase have been associated with AP (Raja et al., 2012; Place et al., 2015; Riley et al., 2021) and prior studies indicated that a cumulative dose of L-asparaginase is a risk factor for disease induction (Chen et al., 2022). The current study have confirmed that both drugs are risk factors for drug-related AP. Thus, it is recommended that long-term high-dose users of these medications be regularly monitored.

Currently, most studies on AP-related protein kinase inhibitors and immune checkpoint inhibitors are case reports. One case of Nilotinib-related AP was reported by Engel et al. (Satoh et al., 2011). This patient was treated with Nilotinib for chronic granulocytic leukemia but developed acute abdominal pain, elevated serum amylase and lipase levels, and enlarged pancreatic parenchyma on enhanced CT 1 day after initiating Nilotinib. All indicators were normal prior to this event, and Nilotinib-related AP, which eventually resolved after discontinuation of the drug, was considered. Kim et al. (Kim et al., 2020) reported a case who presented with persistent abdominal pain, normal serum amylase and lipase levels, and heterogeneous fluid accumulation and fatty infiltration around the tail of the pancreas with no biliary abnormalities 34 days after Lenvatinib treatment. Thus, whether amylase and lipase are elevated or not, AP should be regarded as a differential diagnosis in patients complaining of persistent abdominal pain while using relevant antineoplastic agents. Ueno et al. (Ueno et al., 2021) reported a fatal case of AP induced by Pembrolizumab. In this case, AP was first induced after using Pembrolizumab for 3 months, and the individual was successfully saved by using symptomatic treatment but not discontinued. AP reoccurred 8 months later and failed to be treated successfully, eventually leading to the patient’s death. The timely discontinuation of relevant antineoplastic drugs and symptomatic treatment can be critical to improving AP patient prognosis. Yamamoto et al. (Yamamoto et al., 2021) reported on a 76-year-old patient with malignant melanoma who presented with AP without abdominal pain after administration of Nivolumab and lpilimumab immunotherapy. It is important to identify underlying AP in patients receiving antineoplastic drugs. However, no studies have assessed protein kinase inhibitors and immune checkpoint inhibitors as risk factors for inducing AP. A systematic review and meta-analysis of the incidence of pancreatitis following the use of immune checkpoint inhibitors in patients with advanced cancer found that the incidence of pancreatitis was significantly higher following the use of immune checkpoint inhibitors, with a grade 2 pancreatitis of 1.9% (150/7,702) (George et al., 2019); however, this study did not indicate whether the various immune checkpoint inhibitors were risk factors for inducing drug-related AP. This is the first study to identify antineoplastic drugs as independent risk factors for drug-related AP. These findings can be used to aid the early identification of rare gastrointestinal adverse reactions in patients receiving antineoplastic drugs.

Azathioprine, a prodrug of 6-mercaptopurine, is converted into Mercaptopurine by glutathione S-transferase in the liver. Azathioprine and Mercaptopurine are immunosuppressive drugs that can be used to treat acute leukemia, inflammatory bowel disease, and other immune-related diseases (Nielsen et al., 2001). AP is shown to be a serious adverse event of Azathioprine/Mercaptopurine (Chaparro et al., 2013; Timmer et al., 2016). One Swedish-Danish nationwide cohort study found a significantly increased risk of AP in patients treated with Azathioprine/Mercaptopurine (Wintzell et al., 2019) and another showed that Crohn’s disease patients had a higher risk of Azathioprine-induced AP than those with ulcerative colitis, though the reason remains unknown (Teich et al., 2016). Geenen et al. (van Geenen et al., 2010) found that female Crohn’s disease patients were at particular risk for Azathioprine-related AP (5.1% vs. 0%, *p* = 0.012). Consistent with prior reports, the current study found that Mercaptopurine was a risk factor for drug-related AP. However, the mechanism of AP induced by Azathioprine and 6-mercaptopurine has not been fully elucidated. Broe et al. (Broe and Cameron, 1983) found that Azathioprine significantly affected pancreatic function. Compared with controls, azathioprine administration resulted in a significant increase in secretion and bicarbonate output, and a significant decrease in trypsin output (Broe and Cameron, 1983). In some clinical observations, this depended on the drug dose (Cuffari et al., 1996), but hypersensitivity reactions also seemed to be very important (Ksiądzyna, 2011).

In addition, the current study also found that Ramipril and Lisinopril were risk factors of drug-related AP. Ramipril and Lisinopril are angiotensin-converting enzyme inhibitors (ACEIs). There have been many previous reports of ACEIs induced AP (Gershon and Olshaker, 1998; Masarweh et al., 2023), and the mechanism may be related to localized angioedema in the pancreatic ducts (Griesbacher, 2000; Eland et al., 2006). ACEIs decreased the degradation of bradykinin (bradykinin is associated with the development of angioedema). It has been shown that bradykinin is released during AP, causing the vascular permeability increased and pancreatic edema, which in turn leads to enzymes and other toxic substances trapped in the pancreas, and ultimately pancreatic tissue damage and AP (Griesbacher, 2000; Eland et al., 2006).

Previous studies have identified an association between antibacterial drugs and AP. Case reports suggested that Metronidazole may induce AP (Yilmaz et al., 2016) and a population-based case-control study in Denmark found that Metronidazole increased the risk of AP (<31 days of use [OR: 3; 95% CI:1.4–6.6]; 31–180 days of use [OR:1.8; 95% CI:1.2–2.9]) (Nørgaard et al., 2005). However, stratified analysis showed that only the combination regimen including Metronidazole to treat *Helicobacter pylori* infection was associated with AP (Nørgaard et al., 2005). A population-based case-control study in Sweden found a substantially increased risk of AP within 30 days after the use of oral Metronidazole alone (OR:4.06; 95% CI:1.90–8.64) and in combination with other drugs (OR:11.80; 95% CI: 6.86–20.28) (Barbulescu et al., 2018). The current study also showed that Metronidazole was a risk factor for drug-related AP (OR:1.540; 95% CI:1.340–1.770). Thus, there is sufficient evidence that the use of Metronidazole is related to AP, especially in patients receiving combination drugs for *Helicobacter pylori* infection. These findings highlight a need for increased awareness among physicians. The current study also showed that Tigecycline is a risk factor for drug-related AP (OR:21.400; 95% CI:17.394–26.328), which is consistent with other studies (Hung et al., 2009; Lin et al., 2018; Wang et al., 2021). The mechanism of Tigecycline-induced AP remains unclear, however, this drug is a structural derivative of minocycline, with a chemical structure, pharmacokinetic profile, and adverse effects like those of tetracyclines. Several mechanisms have been proposed for how these drugs induce AP, including the formation of toxic metabolites, hypertriglyceridemia, and elevated bile production (Lin et al., 2018). Interestingly, while 24 of the antibacterial drugs in the current study were associated with drug-related AP, multivariate analysis revealed that only Metronidazole and Tigecycline were risk factors for drug-related AP. This may explain false-positive results reported by individual case reports.

This study found that the antipsychotics, Olanzapine and Quetiapine, were risk factors for drug-related AP. These are second-generation antipsychotics that are linked to metabolic disorders such as hyperglycemia and hypertriglyceridemia (Atmaca et al., 2003). These conditions can cause related complications, such as AP; however, the triggering mechanism remains unclear. This may occur 1) when triglycerides found in chylomicrons are hydrolyzed by pancreatic lipase, leading to the production of free fatty acids and induction of free radical damage (Tsuang et al., 2009) or 2) through an indirect mechanism related to atypical antipsychotics, obesity, and insulin resistance (Yan et al., 2013). Some cases of Olanzapine and Quetiapine-associated AP have been reported (Liou et al., 2014; Baysal et al., 2015). Most antipsychotic-related AP occurs within 3 months (Silva et al., 2016). Thus, drug-related AP should be considered during antipsychotic use, especially in the initial phase.

Eluxadoline, a novel mixture of μ- and κ-opioid receptor agonists and δ-opioid receptor antagonists, was approved in the United States in 2015 for the treatment of diarrhea-predominant irritable bowel syndrome (IBS-D) in adults. Eluxadoline was well tolerated in Phase 2 and Phase 3 trials, with constipation and nausea being the most common adverse effects. While a low incidence of pancreatitis with mild symptoms was also recognized (Dove et al., 2013; Lembo et al., 2016), post-marketing surveillance identified additional cases. Harinstein et al. (Harinstein et al., 2018) used FAERS to identify 119 cases of post-marketing Eluxadoline-associated pancreatitis, including one that resulted in death and 75 in hospitalizations. Subsequent analysis found that most cases occurred in patients without a gallbladder. This finding prompted the FDA to issue a drug safety alert on 15 March 2017, that warned consumers and healthcare-related professionals about the risk of severe pancreatitis that could lead to hospitalization or death associated with Eluxadoline use. On 20 April 2017, the FDA added contraindications to prevent its use in patients without a gallbladder (US Food and Drug Administration, 2017). This study also identified Eluxadoline as a risk factor for drug-related AP (OR:46.255; 95% CI:35.304–60.603). This results may provide valuable information for clinicians.

This study found that the median time to onset of drug-associated AP was 31 days (IQR 7–102 days), with more than 75% of reported cases occurring within the first 100 days after the initiation of drug use. Previous studies of drug-induced AP similarly found that the time interval from initiation to the onset of AP adverse drug reactions was most frequently reported within the first 3 months (Hung et al., 2009; Silva et al., 2016; Harinstein et al., 2018; Wintzell et al., 2019). These findings suggest that the highest risk of drug-related AP is associated with the earliest stages of drug therapy.

This study used data from FAERS, so some limitations should be considered. Firstly, FAERS is based on a self-reporting system and is at risk for underreporting, duplicate reporting, and inaccurate reporting, which can bias research results. Secondly, the lack of information on healthy populations makes it impossible to calculate the incidence of drug-related AP. Thirdly, there is a lack of height data to calculate BMI, making it impossible to define normal weight, overweight, or obese. Fourthly, due to a lack of sufficient data, this study did not take into account particular confounding factors, such as different races, underlying disease, co-medications, or drug doses, which could impact the research results. Fifthly, due to the limitations of the FAERS database, this study did not perform correlation analyses when data were collected from the same locations or countries, which may have led to biased results. Sixthly, the information in this report was not verified, so the monitored signals represent only potential associations. As a result, these findings do not provide the evidence required to confirm a causal relationship between AP and the suspected drugs. Despite these limitations, this was an important exploratory study made in the context of signal mining that highlights the necessity for close monitoring and further investigation using follow-up or case-control studies. This is the most comprehensive study to date that uses FAERS to explore the association between AP and suspected drugs.

## 5 Conclusion

This study used FAERS data to create a comprehensive list of drugs that may be associated with drug-related AP. Individuals within the age range of 41–54 years old, males, or had taken one of 36 drugs, including Tigecycline, were at higher risk for developing drug-related AP. The findings may provide valuable information to aid the early identification of drug-related AP, and inform future studies on the pathogenesis of drug-induced AP. All findings reported here require confirmation using additional clinical studies and animal experiments.

## Data Availability

The original contributions presented in the study are included in the article/Supplementary Material, further inquiries can be directed to the corresponding authors.
