# Supplementary material for: Risk factors for drug-related acute pancreatitis: an analysis of the FDA adverse event reporting system (FAERS)
Source: Front Pharmacol. 2023 Nov 17;14:1231320. doi: 10.3389/fphar.2023.1231320 (PMC10690789; doi:10.3389/fphar.2023.1231320)
Supplement: Supplementary file 1 [file Table1.DOCX]

Supplementary Table S1. Two-by-two contingency table for disproportionality analyses.

|  | Target adverse events | All other adverse events |
| --- | --- | --- |
| Target drug | a | b |
| All other drugs | c | d |
