# Supplementary material for: Risk factors for drug-related acute pancreatitis: an analysis of the FDA adverse event reporting system (FAERS)
Source: Front Pharmacol. 2023 Nov 17;14:1231320. doi: 10.3389/fphar.2023.1231320 (PMC10690789; doi:10.3389/fphar.2023.1231320)
Supplement: Supplementary file 3 [file Table2.DOCX]

Supplementary Table S2. AP-related drugs reported in FAERS.

| **Drug Category** | **Drug** | **Number of Cases** | **ROR** | **95%CI** | **p-value** | **p-adjust** |
| --- | --- | --- | --- | --- | --- | --- |
| Analgesic drugs | Sulindac | 5 | 6.222 | 2.562~15.115 | 0.002 | 1.000 |
|  | Acetaminophen And Tramadol | 29 | 5.543 | 3.836~8.010 | 6.382e-13 | 2.413e-09 |
|  | Nalbuphine | 4 | 5.186 | 1.927~13.961 | 0.008 | 1.000 |
|  | Butalbital And Acetaminophen And Caffeine | 5 | 4.655 | 1.922~11.276 | 0.005 | 1.000 |
|  | Ketoprofen | 16 | 4.360 | 2.659~7.147 | 1.839e-06 | 0.007 |
|  | Acetaminophen And Codeine | 24 | 4.289 | 2.865~6.422 | 7.789e-09 | 2.945e-05 |
|  | Hydrocodone And Acetaminophen | 7 | 3.181 | 1.509~6.704 | 0.008 | 1.000 |
|  | Codeine | 19 | 2.168 | 1.380~3.405 | 0.002 | 1.000 |
|  | Indomethacin | 15 | 2.156 | 1.297~3.585 | 0.006 | 1.000 |
|  | Paracetamol | 96 | 1.888 | 1.544~2.308 | 1.502e-08 | 5.681e-05 |
|  | Tramadol | 150 | 1.583 | 1.348~1.859 | 1.784e-07 | 6.745e-04 |
|  | Acetaminophen | 175 | 1.227 | 1.058~1.424 | 0.008 | 1.000 |
|  | Ibuprofen | 286 | 1.189 | 1.058~1.336 | 0.004 | 1.000 |
| Anti-hypertensive drugs | Trandolapril | 13 | 7.523 | 4.332~13.063 | 4.686e-08 | 1.772e-04 |
|  | Olmesartan Medoxomil | 255 | 6.797 | 6.000~7.699 | 8.786e-119 | 3.322e-115 |
|  | Perindopril And Amlodipine | 4 | 5.967 | 2.213~16.088 | 0.005 | 1.000 |
|  | Candesartan | 39 | 5.153 | 3.753~7.077 | 7.864e-16 | 2.973e-12 |
|  | Lisinopril And Hydrochlorothiazide | 20 | 4.372 | 2.810~6.804 | 9.746e-08 | 3.685e-04 |
|  | Chlorthalidone | 4 | 4.345 | 1.617~11.676 | 0.015 | 1.000 |
|  | Amlodipine And Olmesartan Medoxomil | 11 | 4.237 | 2.334~7.690 | 9.125e-05 | 0.345 |
|  | Olmesartan And Hydrochlorothiazide | 41 | 4.131 | 3.034~5.625 | 1.734e-13 | 6.557e-10 |
|  | Hydrochlorothiazide And Lisinopril | 8 | 3.923 | 1.951~7.888 | 0.001 | 1.000 |
|  | Enalapril | 50 | 3.766 | 2.848~4.979 | 1.360e-14 | 5.141e-11 |
|  | Amlodipine And Benazepril | 14 | 3.740 | 2.206~6.341 | 4.064e-05 | 0.154 |
|  | Perindopril | 25 | 3.331 | 2.245~4.943 | 4.157e-07 | 0.002 |
|  | Losartan And Hydrochlorothiazide | 24 | 3.275 | 2.189~4.900 | 9.307e-07 | 0.004 |
|  | Captopril | 6 | 3.182 | 1.422~7.119 | 0.013 | 1.000 |
|  | Fosinopril | 5 | 2.739 | 1.135~6.614 | 0.039 | 1.000 |
|  | Lisinopril | 229 | 2.628 | 2.306~2.994 | 3.130e-36 | 1.184e-32 |
|  | Losartan | 116 | 2.479 | 2.064~2.977 | 2.001e-17 | 7.567e-14 |
|  | Ramipril | 123 | 2.220 | 1.858~2.651 | 6.077e-15 | 2.298e-11 |
|  | Irbesartan | 44 | 1.910 | 1.420~2.570 | 9.373e-05 | 0.354 |
|  | Eplerenone | 10 | 1.888 | 1.013~3.517 | 0.049 | 1.000 |
|  | Telmisartan | 29 | 1.522 | 1.056~2.192 | 0.029 | 1.000 |
|  | Atenolol | 46 | 1.482 | 1.109~1.980 | 0.011 | 1.000 |
| Anti-osteoporosis drugs | Doxercalciferol | 8 | 5.810 | 2.882~11.713 | 1.026e-04 | 0.388 |
|  | Calcium Acetate | 9 | 3.544 | 1.835~6.843 | 0.001 | 1.000 |
|  | Alendronate And Cholecalciferol | 8 | 2.467 | 1.230~4.951 | 0.018 | 1.000 |
|  | Calcitriol | 10 | 2.467 | 1.323~4.599 | 0.009 | 1.000 |
|  | Pamidronate | 24 | 1.680 | 1.124~2.510 | 0.016 | 1.000 |
|  | Alendronate | 173 | 1.638 | 1.410~1.902 | 2.289e-09 | 8.656e-06 |
| Antiatherosclerotic drugs | Metreleptin | 48 | 16.162 | 12.068~21.644 | 8.723e-40 | 3.298e-36 |
|  | Fenofibrate | 221 | 8.588 | 7.509~9.823 | 2.067e-122 | 7.817e-119 |
|  | Ezetimibe And Simvastatin | 100 | 8.389 | 6.873~10.241 | 1.355e-55 | 5.123e-52 |
|  | Gemfibrozil | 31 | 7.734 | 5.409~11.059 | 1.821e-17 | 6.884e-14 |
|  | Ezetimibe | 270 | 6.968 | 6.172~7.865 | 4.383e-128 | 1.657e-124 |
|  | Simvastatin | 316 | 3.345 | 2.992~3.738 | 4.454e-71 | 1.684e-67 |
|  | Colesevelam | 28 | 2.420 | 1.668~3.511 | 4.048e-05 | 0.153 |
|  | Fluvastatin | 10 | 2.319 | 1.244~4.322 | 0.013 | 1.000 |
|  | Pravastatin | 46 | 2.233 | 1.670~2.985 | 1.300e-06 | 0.005 |
|  | Rosuvastatin | 308 | 2.181 | 1.949~2.441 | 1.674e-33 | 6.328e-30 |
|  | Omega-3 Acid Ethyl Esters | 36 | 2.156 | 1.552~2.993 | 3.754e-05 | 0.142 |
|  | Lovastatin | 7 | 2.106 | 1.001~4.432 | 0.053 | 1.000 |
|  | Atorvastatin | 406 | 1.452 | 1.317~1.602 | 1.623e-12 | 6.138e-09 |
| Antibacterial drugs | Tigecycline | 165 | 24.546 | 20.913~28.809 | 1.456e-159 | 5.505e-156 |
|  | Spiramycine And Metronidazole | 5 | 18.209 | 7.343~45.158 | 1.259e-05 | 0.048 |
|  | Daunorubicin | 24 | 6.358 | 4.240~9.536 | 3.840e-12 | 1.452e-08 |
|  | Cefpodoxime | 8 | 6.211 | 3.079~12.528 | 6.552e-05 | 0.248 |
|  | Doxycycline | 233 | 5.368 | 4.713~6.113 | 4.524e-89 | 1.711e-85 |
|  | Anidulafungin | 5 | 3.775 | 1.561~9.131 | 0.012 | 1.000 |
|  | Cefotaxime | 11 | 3.146 | 1.736~5.702 | 0.001 | 1.000 |
|  | Metronidazole | 144 | 2.907 | 2.466~3.427 | 1.528e-27 | 5.776e-24 |
|  | Terbinafine | 78 | 2.861 | 2.288~3.576 | 2.296e-15 | 8.681e-12 |
|  | Linezolid | 103 | 2.588 | 2.131~3.143 | 6.992e-17 | 2.644e-13 |
|  | Sulfamethoxazole And Trimethoprim | 57 | 2.481 | 1.911~3.221 | 1.953e-09 | 7.383e-06 |
|  | Posaconazole | 25 | 2.480 | 1.672~3.678 | 5.603e-05 | 0.212 |
|  | Cefuroxime | 72 | 2.313 | 1.834~2.918 | 4.444e-10 | 1.680e-06 |
|  | Cefalexin | 13 | 2.295 | 1.329~3.962 | 0.009 | 1.000 |
|  | Micafungin | 16 | 2.053 | 1.255~3.358 | 0.010 | 1.000 |
|  | Amphotericin B | 25 | 1.847 | 1.246~2.738 | 0.004 | 1.000 |
|  | Fluconazole | 54 | 1.834 | 1.403~2.398 | 4.251e-05 | 0.161 |
|  | Minocycline | 30 | 1.788 | 1.248~2.561 | 0.003 | 1.000 |
|  | Telithromycin | 18 | 1.749 | 1.100~2.781 | 0.027 | 1.000 |
|  | Meropenem | 20 | 1.659 | 1.069~2.575 | 0.030 | 1.000 |
|  | Itraconazole | 20 | 1.636 | 1.054~2.539 | 0.043 | 1.000 |
|  | Ceftriaxone | 36 | 1.630 | 1.174~2.262 | 0.005 | 1.000 |
|  | Erythromycin | 23 | 1.524 | 1.012~2.297 | 0.052 | 1.000 |
|  | Amoxicillin And Clavulanate | 73 | 1.375 | 1.092~1.730 | 0.009 | 1.000 |
| Antidiabetic drugs | Alogliptin | 120 | 28.683 | 23.740~34.656 | 5.357e-124 | 2.026e-120 |
|  | Liraglutide | 2492 | 27.905 | 26.754~29.106 | 0.000 | 0.000 |
|  | Sitagliptin | 2833 | 25.940 | 24.935~26.984 | 0.000 | 0.000 |
|  | Linagliptin And Metformin | 40 | 18.807 | 13.636~25.940 | 7.824e-36 | 2.958e-32 |
|  | Saxagliptin | 240 | 18.754 | 16.444~21.389 | 1.572e-205 | 5.945e-202 |
|  | Linagliptin | 455 | 18.626 | 16.927~20.494 | 0.000 | 0.000 |
|  | Alogliptin And Pioglitazone | 10 | 16.082 | 8.483~30.490 | 1.849e-09 | 6.991e-06 |
|  | Sitagliptin And Metformin | 72 | 13.134 | 10.361~16.649 | 6.164e-53 | 2.331e-49 |
|  | Saxagliptin And Metformin | 52 | 12.722 | 9.626~16.812 | 4.475e-38 | 1.692e-34 |
|  | Semaglutide | 503 | 10.508 | 9.608~11.493 | 3.916e-314 | 1.481e-310 |
|  | Exenatide | 2702 | 10.126 | 9.735~10.531 | 0.000 | 0.000 |
|  | Vildagliptin And Metformin | 11 | 8.306 | 4.555~15.147 | 1.926e-07 | 7.282e-04 |
|  | Empagliflozin And Linagliptin | 38 | 8.252 | 5.972~11.401 | 5.750e-22 | 2.174e-18 |
|  | Insulin Degludec And Liraglutide | 19 | 7.977 | 5.051~12.597 | 1.603e-11 | 6.062e-08 |
|  | Dulaglutide | 1348 | 6.997 | 6.624~7.390 | 0.000 | 0.000 |
|  | Nateglinide | 7 | 6.642 | 3.135~14.071 | 1.244e-04 | 0.470 |
|  | Glimepiride | 64 | 6.078 | 4.742~7.789 | 1.224e-28 | 4.627e-25 |
|  | Glibenclamide | 16 | 5.466 | 3.331~8.971 | 1.019e-07 | 3.854e-04 |
|  | Insulin Glargine And Lixisenatide | 31 | 4.735 | 3.318~6.756 | 5.296e-12 | 2.002e-08 |
|  | Dapagliflozin And Metformin | 19 | 4.150 | 2.637~6.531 | 4.316e-07 | 0.002 |
|  | Repaglinide | 16 | 4.090 | 2.495~6.703 | 4.049e-06 | 0.015 |
|  | Canagliflozin | 347 | 4.002 | 3.598~4.451 | 2.703e-97 | 1.022e-93 |
|  | Metformin | 818 | 3.986 | 3.718~4.273 | 1.219e-224 | 4.609e-221 |
|  | Empagliflozin | 288 | 3.797 | 3.379~4.267 | 2.790e-76 | 1.055e-72 |
|  | Dapagliflozin | 201 | 3.689 | 3.209~4.241 | 4.878e-52 | 1.844e-48 |
|  | Canagliflozin And Metformin | 10 | 2.663 | 1.428~4.966 | 0.006 | 1.000 |
|  | Empagliflozin And Metformin | 11 | 2.328 | 1.286~4.216 | 0.010 | 1.000 |
|  | Albiglutide | 76 | 2.235 | 1.783~2.801 | 5.400e-10 | 2.042e-06 |
| Antiepileptic drugs | Valproic Acid | 643 | 5.615 | 5.191~6.074 | 1.241e-251 | 4.691e-248 |
|  | Rufinamide | 6 | 5.037 | 2.245~11.303 | 0.002 | 1.000 |
| Antineoplastic drugs | L-Asparaginase | 254 | 23.662 | 20.799~26.919 | 8.871e-241 | 3.354e-237 |
|  | Pegaspargase | 228 | 17.852 | 15.604~20.425 | 6.380e-191 | 2.412e-187 |
|  | Ponatinib | 167 | 6.009 | 5.153~7.007 | 4.409e-71 | 1.667e-67 |
|  | Tioguanine | 9 | 5.912 | 3.052~11.452 | 3.382e-05 | 0.128 |
|  | Clofarabine | 27 | 5.048 | 3.448~7.390 | 2.996e-11 | 1.133e-07 |
|  | Nilotinib | 419 | 4.750 | 4.311~5.234 | 9.569e-141 | 3.618e-137 |
|  | Brentuximab Vedotin | 79 | 4.302 | 3.444~5.374 | 1.698e-25 | 6.421e-22 |
|  | Lenvatinib | 181 | 3.782 | 3.265~4.381 | 1.764e-48 | 6.669e-45 |
|  | Porfimer | 4 | 3.280 | 1.223~8.796 | 0.036 | 1.000 |
|  | Arsenic Trioxide | 13 | 3.269 | 1.891~5.650 | 2.722e-04 | 1.000 |
|  | Sunitinib | 14 | 3.247 | 1.916~5.501 | 1.730e-04 | 0.654 |
|  | Inotuzumab Ozogamicin | 8 | 2.802 | 1.396~5.626 | 0.009 | 1.000 |
|  | Durvalumab | 58 | 2.527 | 1.951~3.273 | 8.011e-10 | 3.029e-06 |
|  | Vincristine | 56 | 2.449 | 1.882~3.187 | 6.297e-09 | 2.381e-05 |
|  | Gemtuzumab Ozogamicin | 16 | 2.387 | 1.459~3.906 | 0.002 | 1.000 |
|  | Tamoxifen | 33 | 2.183 | 1.549~3.075 | 5.286e-05 | 0.200 |
|  | Bosutinib | 44 | 2.174 | 1.616~2.926 | 3.778e-06 | 0.014 |
|  | Cobimetinib | 12 | 2.130 | 1.207~3.761 | 0.017 | 1.000 |
|  | Trametinib | 60 | 2.071 | 1.606~2.670 | 3.804e-07 | 0.001 |
|  | Ipilimumab | 109 | 1.961 | 1.624~2.368 | 2.677e-10 | 1.012e-06 |
|  | Vemurafenib | 68 | 1.949 | 1.535~2.474 | 6.603e-07 | 0.002 |
|  | Neratinib | 11 | 1.896 | 1.047~3.431 | 0.055 | 1.000 |
|  | Cytarabine | 61 | 1.869 | 1.452~2.404 | 9.480e-06 | 0.036 |
|  | Hydroxyurea | 21 | 1.828 | 1.190~2.809 | 0.011 | 1.000 |
|  | Sorafenib | 113 | 1.827 | 1.518~2.198 | 5.286e-09 | 1.999e-05 |
|  | Tretinoin | 14 | 1.815 | 1.073~3.071 | 0.043 | 1.000 |
|  | Atezolizumab | 94 | 1.773 | 1.447~2.172 | 4.188e-07 | 0.002 |
|  | Pembrolizumab | 199 | 1.769 | 1.538~2.034 | 2.448e-13 | 9.256e-10 |
|  | Regorafenib | 52 | 1.678 | 1.277~2.204 | 5.735e-04 | 1.000 |
|  | Nivolumab | 360 | 1.669 | 1.504~1.852 | 4.865e-19 | 1.839e-15 |
|  | Epirubicin | 25 | 1.616 | 1.091~2.395 | 0.021 | 1.000 |
|  | Melphalan | 30 | 1.484 | 1.037~2.125 | 0.044 | 1.000 |
|  | Doxorubicin | 127 | 1.378 | 1.157~1.641 | 5.601e-04 | 1.000 |
|  | Irinotecan | 57 | 1.314 | 1.012~1.704 | 0.047 | 1.000 |
|  | Bevacizumab | 247 | 1.151 | 1.016~1.305 | 0.031 | 1.000 |
| Antipsychotic drugs | Quetiapine | 3322 | 14.576 | 14.062~15.108 | 0.000 | 0.000 |
|  | Olanzapine | 1399 | 10.298 | 9.756~10.870 | 0.000 | 0.000 |
|  | Fluphenazine | 5 | 5.431 | 2.239~13.174 | 0.003 | 1.000 |
|  | Olanzapine And Fluoxetine | 8 | 3.416 | 1.700~6.863 | 0.003 | 1.000 |
|  | Mirtazapine | 70 | 1.334 | 1.055~1.688 | 0.019 | 1.000 |
|  | Aripiprazole | 247 | 1.151 | 1.015~1.304 | 0.031 | 1.000 |
| Antirheumatic drugs | Brodalumab | 10 | 3.388 | 1.815~6.324 | 0.001 | 1.000 |
|  | Tocilizumab | 268 | 1.525 | 1.352~1.720 | 1.138e-10 | 4.304e-07 |
|  | Infliximab | 676 | 1.358 | 1.258~1.465 | 5.231e-14 | 1.978e-10 |
| Antituberculosis drugs | Pyrazinamide | 4 | 6.927 | 2.565~18.710 | 0.003 | 1.000 |
|  | Isoniazid | 56 | 6.446 | 4.943~8.405 | 1.829e-26 | 6.915e-23 |
|  | Rifampicin And Isoniazid And Pyrazimide | 5 | 5.203 | 2.146~12.615 | 0.003 | 1.000 |
|  | Rifampicin | 74 | 4.731 | 3.758~5.955 | 2.347e-26 | 8.873e-23 |
|  | Bedaquiline | 18 | 2.843 | 1.786~4.525 | 1.157e-04 | 0.437 |
| Antiviral drugs | Didanosine | 90 | 24.102 | 19.409~29.930 | 2.852e-87 | 1.078e-83 |
|  | Stavudine | 47 | 10.875 | 8.119~14.565 | 1.017e-31 | 3.844e-28 |
|  | Emtricitabine | 14 | 6.866 | 4.037~11.678 | 4.323e-08 | 1.635e-04 |
|  | Enfuvirtide | 27 | 5.429 | 3.707~7.950 | 6.139e-12 | 2.321e-08 |
|  | Indinavir | 8 | 5.173 | 2.568~10.419 | 2.217e-04 | 0.838 |
|  | Fosamprenavir | 10 | 4.754 | 2.542~8.888 | 7.530e-05 | 0.285 |
|  | Tipranavir | 4 | 4.672 | 1.738~12.565 | 0.012 | 1.000 |
|  | Etravirine | 11 | 3.294 | 1.817~5.972 | 7.317e-04 | 1.000 |
|  | Abacavir | 37 | 3.254 | 2.352~4.501 | 1.569e-09 | 5.931e-06 |
|  | Darunavir | 40 | 3.144 | 2.301~4.295 | 9.165e-10 | 3.465e-06 |
|  | Raltegravir | 62 | 3.135 | 2.440~4.029 | 3.327e-14 | 1.258e-10 |
|  | Foscarnet | 7 | 3.052 | 1.448~6.431 | 0.009 | 1.000 |
|  | Atazanavir | 47 | 3.042 | 2.281~4.056 | 9.700e-11 | 3.668e-07 |
|  | Lopinavir And Ritonavir | 50 | 2.788 | 2.109~3.684 | 4.531e-10 | 1.713e-06 |
|  | Dolutegravir | 44 | 2.738 | 2.034~3.686 | 7.887e-09 | 2.982e-05 |
|  | Ombitasvir And Paritaprevir | 34 | 2.727 | 1.944~3.823 | 3.962e-07 | 0.001 |
|  | Emtricitabine And Tenofovir Alafenamide | 13 | 2.460 | 1.424~4.248 | 0.003 | 1.000 |
|  | Odefsey | 7 | 2.448 | 1.163~5.155 | 0.027 | 1.000 |
|  | Peginterferon Alfa-2b | 110 | 2.353 | 1.950~2.840 | 3.883e-15 | 1.468e-11 |
|  | Abacavir And Lamivudine | 41 | 2.301 | 1.692~3.130 | 2.241e-06 | 0.008 |
|  | Lamivudine And Zidovudine | 22 | 2.287 | 1.503~3.480 | 5.038e-04 | 1.000 |
|  | Nevirapine | 53 | 2.261 | 1.725~2.964 | 1.362e-07 | 5.150e-04 |
|  | Genvoya | 20 | 2.231 | 1.437~3.466 | 0.001 | 1.000 |
|  | Ganciclovir | 10 | 2.186 | 1.173~4.074 | 0.029 | 1.000 |
|  | Telaprevir | 189 | 2.139 | 1.853~2.469 | 2.454e-20 | 9.277e-17 |
|  | Biktarvy | 26 | 2.123 | 1.443~3.123 | 4.902e-04 | 1.000 |
|  | Ritonavir | 46 | 1.995 | 1.493~2.667 | 2.342e-05 | 0.089 |
|  | Entecavir | 30 | 1.862 | 1.300~2.667 | 0.002 | 1.000 |
|  | Simeprevir | 16 | 1.793 | 1.096~2.932 | 0.027 | 1.000 |
|  | Ribavirin | 112 | 1.793 | 1.488~2.159 | 1.917e-08 | 7.248e-05 |
|  | Lamivudine | 54 | 1.791 | 1.370~2.340 | 7.864e-05 | 0.297 |
|  | Efavirenz | 29 | 1.769 | 1.227~2.549 | 0.004 | 1.000 |
|  | Boceprevir | 32 | 1.735 | 1.225~2.456 | 0.003 | 1.000 |
|  | Aciclovir | 56 | 1.704 | 1.310~2.217 | 2.199e-04 | 0.832 |
|  | Tenofovir | 14 | 1.693 | 1.001~2.864 | 0.054 | 1.000 |
|  | Remdesivir | 39 | 1.615 | 1.179~2.213 | 0.006 | 1.000 |
| Diuretic drugs | Metolazone | 10 | 8.120 | 4.325~15.246 | 8.357e-07 | 0.003 |
|  | Hydrochlorothiazide | 176 | 7.214 | 6.208~8.382 | 1.780e-86 | 6.731e-83 |
|  | Torasemide | 16 | 3.286 | 2.006~5.381 | 5.403e-05 | 0.204 |
|  | Furosemide | 104 | 1.572 | 1.296~1.907 | 1.534e-05 | 0.058 |
| Gastric acid secretion inhibitors | Esomeprazole | 55 | 3.305 | 2.533~4.313 | 1.173e-13 | 4.437e-10 |
|  | Omeprazole | 212 | 2.307 | 2.015~2.642 | 1.793e-26 | 6.778e-23 |
|  | Rabeprazole | 27 | 2.191 | 1.500~3.200 | 2.654e-04 | 1.000 |
|  | Pantoprazole | 188 | 1.921 | 1.664~2.218 | 6.967e-16 | 2.634e-12 |
| Glucocorticoids | Betamethasone | 25 | 3.019 | 2.035~4.479 | 2.295e-06 | 0.009 |
|  | Prednisone | 156 | 1.756 | 1.500~2.056 | 1.364e-10 | 5.159e-07 |
|  | Budesonide | 76 | 1.615 | 1.289~2.024 | 1.002e-04 | 0.379 |
|  | Dexamethasone | 149 | 1.414 | 1.204~1.662 | 6.016e-05 | 0.227 |
|  | Prednisolone | 125 | 1.281 | 1.074~1.528 | 0.007 | 1.000 |
| Immunomodulatory drugs | Mercaptopurine | 118 | 14.951 | 12.414~18.007 | 1.494e-91 | 5.648e-88 |
|  | Azathioprine | 212 | 11.822 | 10.298~13.572 | 1.976e-143 | 7.473e-140 |
|  | Interferon Alfacon-1 | 8 | 5.776 | 2.865~11.644 | 1.067e-04 | 0.404 |
|  | Antithymocyte Globulin Rabbit | 7 | 3.922 | 1.859~8.276 | 0.003 | 1.000 |
|  | Basiliximab | 20 | 3.629 | 2.334~5.644 | 1.629e-06 | 0.006 |
|  | Interferon Alfa-2b | 26 | 2.276 | 1.547~3.348 | 1.641e-04 | 0.620 |
|  | Thymoglobuline | 34 | 2.268 | 1.618~3.179 | 2.264e-05 | 0.086 |
|  | Peg-Interferon Alfa 2a | 14 | 1.863 | 1.101~3.152 | 0.027 | 1.000 |
|  | Mycophenolate | 216 | 1.640 | 1.434~1.875 | 1.955e-11 | 7.391e-08 |
|  | Tacrolimus | 218 | 1.331 | 1.165~1.521 | 5.369e-05 | 0.203 |
|  | Leflunomide | 73 | 1.306 | 1.037~1.644 | 0.027 | 1.000 |
| Others | Liposyn | 5 | 112.568 | 39.110~323.997 | 4.430e-09 | 1.675e-05 |
|  | Eluxadoline | 348 | 48.909 | 43.587~54.881 | 0.000 | 0.000 |
|  | Iothalamate Meglumine | 6 | 20.638 | 8.973~47.465 | 8.483e-07 | 0.003 |
|  | Puregon Follitropin Beta | 4 | 17.079 | 6.200~47.045 | 1.212e-04 | 0.458 |
|  | Riluzole | 55 | 14.379 | 10.955~18.873 | 8.312e-43 | 3.143e-39 |
|  | Dicyclomine | 13 | 8.894 | 5.115~15.468 | 7.048e-09 | 2.665e-05 |
|  | Mesalazine | 335 | 8.660 | 7.764~9.659 | 1.968e-185 | 7.441e-182 |
|  | Carglumic Acid | 13 | 8.496 | 4.887~14.768 | 1.188e-08 | 4.493e-05 |
|  | Calcium Carbonate | 20 | 8.340 | 5.341~13.023 | 2.198e-12 | 8.310e-09 |
|  | Balsalazide | 8 | 7.420 | 3.673~14.993 | 1.938e-05 | 0.073 |
|  | Givosiran | 8 | 6.739 | 3.339~13.603 | 3.761e-05 | 0.142 |
|  | Thiamazole | 27 | 4.836 | 3.304~7.079 | 7.528e-11 | 2.846e-07 |
|  | Clomiphene | 11 | 4.697 | 2.587~8.530 | 3.729e-05 | 0.141 |
|  | Lisdexamfetamine | 4 | 4.695 | 1.746~12.625 | 0.012 | 1.000 |
|  | Ivacaftor | 70 | 4.661 | 3.679~5.906 | 1.130e-24 | 4.272e-21 |
|  | Levonorgestrel And Ethinylestradiol | 25 | 3.816 | 2.570~5.664 | 3.528e-08 | 1.334e-04 |
|  | Propofol | 69 | 3.501 | 2.760~4.441 | 6.290e-18 | 2.378e-14 |
|  | Pylera | 7 | 3.340 | 1.584~7.042 | 0.006 | 1.000 |
|  | Soybean Oil | 6 | 3.182 | 1.422~7.119 | 0.013 | 1.000 |
|  | Cyanocobalamin | 4 | 3.125 | 1.166~8.378 | 0.042 | 1.000 |
|  | Drospirenone And Ethinylestradiol | 427 | 3.053 | 2.774~3.360 | 9.992e-84 | 3.778e-80 |
|  | Ethiodized Oil | 6 | 3.051 | 1.364~6.825 | 0.016 | 1.000 |
|  | Teduglutide | 90 | 2.845 | 2.311~3.503 | 2.489e-17 | 9.411e-14 |
|  | Lubiprostone | 14 | 2.726 | 1.610~4.616 | 9.319e-04 | 1.000 |
|  | Pentoxifylline | 4 | 2.699 | 1.008~7.230 | 0.064 | 1.000 |
|  | Atovaquone And Proguanil | 17 | 2.653 | 1.645~4.279 | 3.828e-04 | 1.000 |
|  | Desloratadine | 17 | 2.614 | 1.621~4.215 | 4.513e-04 | 1.000 |
|  | Lanthanum Carbonate | 19 | 2.508 | 1.596~3.942 | 3.553e-04 | 1.000 |
|  | Anagrelide | 11 | 2.409 | 1.330~4.362 | 0.008 | 1.000 |
|  | Calcium Folinate | 7 | 2.381 | 1.131~5.013 | 0.031 | 1.000 |
|  | Memantine | 75 | 2.258 | 1.799~2.835 | 5.504e-10 | 2.081e-06 |
|  | Colchicine | 19 | 2.211 | 1.408~3.474 | 0.002 | 1.000 |
|  | Alfuzosin | 13 | 2.148 | 1.244~3.708 | 0.012 | 1.000 |
|  | Aprepitant | 21 | 1.999 | 1.301~3.071 | 0.005 | 1.000 |
|  | Allopurinol | 54 | 1.988 | 1.521~2.599 | 4.888e-06 | 0.018 |
|  | Loperamide | 73 | 1.955 | 1.552~2.461 | 2.739e-07 | 0.001 |
|  | Roflumilast | 15 | 1.945 | 1.170~3.233 | 0.017 | 1.000 |
|  | Icodextrin | 43 | 1.819 | 1.347~2.455 | 2.858e-04 | 1.000 |
|  | Acitretin | 12 | 1.793 | 1.016~3.165 | 0.050 | 1.000 |
|  | Trikafta | 42 | 1.602 | 1.182~2.170 | 0.004 | 1.000 |
|  | Orlistat | 141 | 1.538 | 1.303~1.815 | 1.778e-06 | 0.007 |
|  | Dronedarone | 28 | 1.510 | 1.041~2.189 | 0.035 | 1.000 |
|  | Vedolizumab | 197 | 1.369 | 1.190~1.575 | 2.834e-05 | 0.107 |
|  | Epoprostenol | 58 | 1.346 | 1.039~1.742 | 0.027 | 1.000 |
|  | Nintedanib | 66 | 1.309 | 1.028~1.668 | 0.034 | 1.000 |
|  | Deferasirox | 98 | 1.306 | 1.071~1.593 | 0.011 | 1.000 |
|  | Dialysis Intraperitoneal | 659 | 1.250 | 1.157~1.350 | 3.885e-08 | 1.469e-04 |

AP, acute pancreatitis; ROR, reporting odds ratio; CI, confidence interval; FAERS, FDA Adverse Event Reporting System; P-adjust, p-value after Bonferroni correction; P-adjust<0.01, statistically significant.
