# Supplementary material for: Risk factors for drug-related acute pancreatitis: an analysis of the FDA adverse event reporting system (FAERS)
Source: Front Pharmacol. 2023 Nov 17;14:1231320. doi: 10.3389/fphar.2023.1231320 (PMC10690789; doi:10.3389/fphar.2023.1231320)
Supplement: Supplementary file 4 [file Table3.DOCX]

Supplementary Table S3. The categories of 36 drugs.

| **Drug Category** | **Drug** | **OR** | **95%CI** | **p-value** | **p-adjust** |
| --- | --- | --- | --- | --- | --- |
| Anti-hypertensive drugs | Ramipril | 1.318 | 1.195~1.454 | 3.21e-08 | 1.45e-06 |
|  | Lisinopril | 1.161 | 1.087~1.240 | 8.94e-06 | 4.02e-04 |
| Antiatherosclerotic drugs | Fenofibrate | 2.717 | 2.481~2.975 | 1.51e-103 | 6.78e-102 |
|  | Ezetimibe | 1.659 | 1.475~1.868 | 4.34e-17 | 1.96e-15 |
| Antibacterial drugs | Tigecycline | 21.400 | 17.394~26.328 | 1.57e-184 | 7.07e-183 |
|  | Metronidazole | 1.540 | 1.340~1.770 | 1.25e-09 | 5.61e-08 |
| Antidiabetic drugs | Liraglutide | 16.538 | 15.387~17.774 | 0.000 | 0.000 |
|  | Semaglutide | 7.385 | 6.194~8.806 | 6.15e-110 | 2.77e-108 |
|  | Dulaglutide | 7.034 | 6.104~8.105 | 3.66e-160 | 1.65e-158 |
|  | Alogliptin | 6.369 | 4.881~8.311 | 2.39e-42 | 1.08e-40 |
|  | Exenatide | 4.217 | 3.930~4.526 | 0.000 | 0.000 |
|  | Sitagliptin | 3.411 | 3.147~3.697 | 1.78e-195 | 8.03e-194 |
|  | Saxagliptin | 3.280 | 2.606~4.130 | 5.03e-24 | 2.26e-22 |
|  | Linagliptin | 2.778 | 2.312~3.338 | 1.04e-27 | 4.69e-26 |
|  | Metformin | 1.291 | 1.223~1.362 | 1.29e-20 | 5.79e-19 |
| Antiepileptic drugs | Valproic Acid | 1.799 | 1.635~1.979 | 1.98e-33 | 8.93e-32 |
| Antineoplastic drugs | Pegaspargase | 8.556 | 7.194~10.175 | 3.82e-130 | 1.72e-128 |
|  | Ponatinib | 7.639 | 6.035~9.670 | 3.91e-64 | 1.76e-62 |
|  | L-Asparaginase | 5.377 | 4.299~6.726 | 3.98e-49 | 1.79e-47 |
|  | Nilotinib | 3.454 | 2.700~4.419 | 6.10e-23 | 2.75e-21 |
|  | Lenvatinib | 2.132 | 1.687~2.694 | 2.24e-10 | 1.01e-08 |
|  | Sorafenib | 2.106 | 1.590~2.789 | 2.07e-07 | 9.30e-06 |
|  | Nivolumab | 1.961 | 1.644~2.339 | 7.59e-14 | 3.42e-12 |
|  | Pembrolizumab | 1.885 | 1.559~2.281 | 6.62e-11 | 2.98e-09 |
| Antipsychotic drugs | Olanzapine | 5.677 | 5.278~6.107 | 0.000 | 0.000 |
|  | Quetiapine | 3.806 | 3.569~4.059 | 0.000 | 0.000 |
| Antiviral drugs | Ribavirin | 1.680 | 1.451~1.944 | 3.91e-12 | 1.76e-10 |
| Diuretic drugs | Hydrochlorothiazide | 1.395 | 1.279~1.522 | 5.25e-14 | 2.36e-12 |
| Gastric acid secretion inhibitors | Pantoprazole | 1.402 | 1.310~1.500 | 9.91e-23 | 4.46e-21 |
| Immunomodulatory drugs | Azathioprine | 2.326 | 2.032~2.663 | 2.32e-34 | 1.05e-32 |
|  | Mercaptopurine | 2.174 | 1.803~2.620 | 3.83e-16 | 1.72e-14 |
|  | Mycophenolate | 1.464 | 1.234~1.737 | 1.23e-05 | 5.53e-04 |
| Others | Eluxadoline | 46.255 | 35.304~60.603 | 2.86e-170 | 1.29e-168 |
|  | Drospirenone And Ethinylestradiol | 3.202 | 2.850~3.599 | 3.56e-85 | 1.60e-83 |
|  | Orlistat | 3.108 | 2.414~4.001 | 1.44e-18 | 6.47e-17 |
|  | Mesalazine | 1.915 | 1.674~2.191 | 2.86e-21 | 1.29e-19 |

CI, confidence interval; OR, odds ratio; P-adjust, p-value after Bonferroni correction; P-adjust<0.01, statistically significant.
